# Negative Energy Balance Enhances Ultradian Rhythmicity in Spring-Programmed Voles

**DOI:** 10.1177/07487304211005640

**Published:** 2021-04-20

**Authors:** Laura van Rosmalen, Roelof A. Hut

**Affiliations:** Chronobiology Unit, Groningen Institute for Evolutionary Life Sciences, University of Groningen, Groningen, the Netherlands

**Keywords:** circadian rhythms, ultradian rhythms, food deprivation, temperature, metabolism, negative energy balance, *Microtus arvalis*, *Microtus oeconomus*

## Abstract

Voles are small herbivorous rodents that can display both circadian activity rhythms (~24-h periodicity) and ultradian activity rhythms (~1- to 6-h periodicity). Ultradian rhythms are observed on an individual level, but also in synchronized populations. Ultradian rhythm period has been suggested to be influenced by energy balance, but the underlying mechanisms of ultradian rhythmicity are poorly understood. We manipulated energy balance by implementing the “work-for-food” paradigm, in which small rodents are exposed to increasing levels of food scarcity at different ambient temperatures in the laboratory. Photoperiodical spring-programmed voles on high workload changed their nocturnal circadian activity and body temperature rhythm to ultradian patterns, indicating that a negative energy balance induces ultradian rhythmicity. This interpretation was confirmed by the observation that ultradian patterns arose earlier at low temperatures. Interestingly, a positive relationship between ultradian period length and workload was observed in tundra voles. Spectral analysis revealed that the power of ultradian rhythmicity increased at high workload, whereas the circadian component of running wheel activity decreased. This study shows that the balance between circadian and ultradian rhythmicity is determined by energy balance, confirming flexible circadian and ultradian rhythms in females and males of 2 different vole species: the common vole (*Microtus arvalis*) and the tundra vole (*Microtus oeconomus*).

Activity patterns in small mammals can be assigned to circadian (~24-h period) and ultradian (1- to 6-h period) rhythms. The suprachiasmatic nucleus (SCN) of the hypothalamus is the circadian pacemaker entrained by light and responsible for the circadian organization of physiology and behavior (Moore and Eichler, 1972; Ralph et al., 1990; Stephan and Zucker, 1972). Microtine rodents, such as voles, are characterized by their ultradian locomotor activity and feeding rhythms (Gerkema et al., 1990; Halle, 1995; Korslund, 2006; Nieminen et al., 2013). Synchronization of ultradian activity among individuals may reduce individual predation risk due to safety in numbers (Daan and Slopsema, 1978), by confusing the predator and as a cue to exchange warning signals (Gerkema and Verhulst, 1990).

SCN-lesioned common voles lose their circadian rhythmicity like other small rodents, while ultradian rhythmicity in behavior is sustained (Gerkema et al., 1990). Because short-period rhythms are free-running when animals are exposed to constant darkness (DD), an endogenous clock mechanism must be assigned to be responsible for generating ultradian rhythms (Daan and Slopsema, 1978; Gerkema et al., 1993). Although the neuronal brain networks and origin of ultradian rhythms are largely unknown, synchronous ultradian calcium rhythms in the paraventricular nucleus (PVN) and subparaventricular zone (SPZ) in the hypothalamus have recently been observed (Wu et al., 2018).

Long-term recordings of vole activity revealed that ultradian rhythms are a seasonally occurring phenomena in autumn and winter (Daan and Slopsema, 1978; Erkinaro, 1961; Halle, 1995). This suggests that photoperiod may be the driving factor for ultradian rhythmicity. However, the positive correlation between the period length of ultradian rhythms and body mass, both among (Daan and Slopsema, 1978) and within species (Gerkema and Daan, 1985), suggests that ultradian patterns are related to the metabolic demands of an animal. Although food deprivation does not change the ultradian period in voles (Gerkema and van der Leest, 1991), low ambient temperatures increase O_2_ consumption and shorten the period of the ultradian feeding cycle (Daan and Slopsema, 1978).

A systematic understanding of how energy balance contributes to the period of ultradian rhythms is still lacking. By manipulating both food availability and ambient temperature, we investigated the relationship between energy balance and the ultradian rhythm period. By offering voles access to a running wheel, both the circadian and ultradian components of behavior can be studied (van der Veen et al., 2006). The energy balance of small rodents can be manipulated without restricting access to food at a specific time of day in the laboratory using the “work-for-food” paradigm (Hut et al., 2011; van der Vinne et al., 2014, 2015 a, 2015b). In this paradigm, we induce a natural food scarcity in the laboratory by changing the amount of wheel rotations that an animal has to make to obtain a food pellet.

We hypothesize that a negative energy balance will arise in animals on a high workload and drives ultradian rhythmicity, while the circadian component becomes weaker. Furthermore, ultradian period length is expected to decrease with an increasing negative energy balance. Also, the amplitude of core body temperature rhythms is expected to increase under food scarcity by lowered body temperatures during rest, resulting in reduced energy loss when animals are inactive. This study was undertaken to assess how energy balance information (food, fat and temperature) shapes activity patterns in two different vole species: the common vole (*Microtus arvalis*) and the tundra vole (*Microtus oeconomus*).

## Materials and methods

### Animals, Logger Implantation, and Experimental Design

All experimental procedures were carried out according to the guidelines of the animal welfare body (IvD) of the University of Groningen, and all experiments were approved by the Centrale Commissie Dierproeven of the Netherlands (CCD, license number: AVD1050020186147). Common voles (*M. arvalis*) were obtained from the Lauwersmeer area (Netherlands, 53°24′N, 6°16′E, (Gerkema et al., 1993). Occasionally voles from natural common vole populations have been added to the colony to prevent the lab population from inbreeding. Tundra voles (*M. oeconomus*) were obtained from 4 different regions in the Netherlands (described in van de Zande et al., 2000). Both populations were kept successfully in the lab as outbred colonies, and all voles in this study were indoor-bred at the University of Groningen. The experiments were carried out in climate chambers with 55% ± 5% relative humidity.

Voles (common voles: 31 females and 34 males; tundra voles: 29 females and 39 males) were born under a short photoperiod (SP, 8-h light:16-h dark) at 21 ± 1 °C and weaned at an age of 21 days. At weaning, animals were housed in individual cages (15 × 32 × 13 cm^3^) and exposed to a long photoperiod (common voles: LP, 16-h light:8-h dark; tundra voles: LP, 14-h light:10-h dark) at either 21 ± 1 °C or 10 ± 1 °C. The transition from SP to LP mimics spring, during which circadian activity patterns are prominently observed in nature (Daan and Slopsema, 1978; Erkinaro, 1961; Halle, 1995). To assess core body temperature patterns, 1.96 g thermologgers (AM100 WeePit; Alpha Mach Inc., Sainte-Julie, QC, Canada; sample interval, 10 min) were implanted in the abdominal cavity of a subset of animals when 28 days old. Thermologgers were kept in saline with penicillin just before surgery. Animals were operated under isoflurane anesthesia (2%-3%). Directly after surgery, a subcutaneous injection of 4 mg/kg Finadyne was administrated as postoperative analgesic. Animals were closely monitored and weighed during the recovery period.

All animals were transferred to cages (15 × 32 × 13 cm^3^) provided with running wheels (14 cm diameter) when 35 days old and assigned to either a high or a low workload schedule at day 40. Experiments were completed when 75 days old. Running wheel activity was recorded continuously and stored in 1-min bins. Ad libitum, food was available for all animals until 40 days old. Voles were provided with water ad libitum throughout the course of the experiments.

### Work-for-Food Protocol

In the work-for-food protocol, animals had to make a set number of wheel revolutions (42.8 cm per revolution) to receive a 45-mg food pellet (630 J per pellet) (F0165; Bio-Serv, Flemington NJ, USA), using a computer-controlled food dispenser (Med Associates Inc., St. Albans VT, USA). All animals started on a low workload protocol (100 revolutions/pellet = 0.07 m/J). This is similar to ad libitum food conditions, since there were always remaining food pellets left in the cages of animals under a low workload. Half of the animals were exposed to an increasing workload paradigm in which workload was increased daily by an additional 10-30 revolutions per pellet to induce a negative energy balance over time. The gradual increase in workload at different ambient temperatures allows voles to be active and receive food at all times of day. The number of wheel rotations a day, and therefore the number of food pellets received, strongly depend on the animal and experimental condition. Therefore, the increase in workload per day was determined based on individual body mass and the amount of earned food pellets. Workload was not increased for animals that were losing weight too fast, whereas workload was increased further for animals that were not losing weight yet. At the end of the paradigm, higher workloads were achieved in experimental groups at 21 °C. All voles were weighed every other day throughout the course of the experiments to monitor body mass loss and to keep animals above 75% of their initial body mass (35 days old).

### Activity and Body Temperature Analysis

Running wheel activity data were recorded in 1-min bins. Actograms were generated using Actoview for MS Excel 2010 (programmed by C. Mulder, University of Groningen). Five-day average activity profiles were generated for each group (Suppl. Figs. S2 and S3). To assess the period of activity rhythms, a Morlet wavelet time-series analysis was performed using the R-package “WaveletComp” (Rösch and Schmidbauer, 2016). One-hour bin activity data were used to assess the wavelet power of the activity period. To assess the relationship between workload/body mass and ultradian period length (Figs. 3g, 3h and 4g, 4h and Suppl. Fig. S5), we are exclusively interested in the ultradian component. Therefore, we extracted the ultradian component by taking the maximum wavelet power (if ≥0.1) of the activity period <5 h for each animal for each day to generate Figures 3g, 3h and 4g, 4h and Suppl. Fig. S5. Body temperature data were recorded in 10-min bins, and 5-day average temperature profiles were generated for each group. Body temperature heatmaps were generated using the R-package “gplots” (Warnes et al., 2015).

### Statistical Analysis

All analyses were performed using RStudio (version 1.2.1335) (R Core Team, 2013), and figures were generated using the R-package “ggplot2” (Wickham, 2016). The effects of workload on body mass, food intake, and different parameters of activity and core body temperature patterns were assessed by repeated-measures linear mixed-effects models, using the R-package “lme4” (Bates et al., 2014). Stepwise backward elimination was used, and independent variables with nonsignificant *p*-values were omitted for final models (Suppl. Table S1). When the interaction term was significant, the underlying single terms were kept in the model, even when nonsignificant. For contrast analysis, separate models for common and tundra voles were created, and pairwise comparisons were performed using a post hoc Tukey honestly significant difference (HSD) test. Statistical significance was determined at *p* < 0.05.

## Results

### Energy Balance Can Be Manipulated by the Work-for-Food Protocol and Ambient Temperature

To test the prediction that a negative energy balance enhances ultradian rhythmicity in voles, we manipulated food availability and ambient temperature. Letting voles work for their food at gradually increasing workload levels at different ambient temperatures reduced energy intake by 10%-70% (*F*_1,4413_ = 1308.92, *p* < 0.0001; Suppl. Fig. S4C and S4D) without restricting food access to a specific time of day. Energy intake was 30% higher at 10° C than at 21° C in common voles on high workload (0.25  m/J) (males: *p* < 0.0001; females: *p* < 0.0001; Suppl. Fig. S4C). However, in tundra voles, energy intake was not affected by temperature (*F*_1,65_ = 0.03, ns). Increasing workload induced a 6-fold increase in total daily wheel running activity (*F*_1,4199_ = 3859.23, *p* < 0.0001; Suppl. Fig. S4A and S4B). Female common voles showed significantly higher baseline activity levels than male common voles (*F*_1,3581_ = 57.11, *p* < 0.0001; Suppl. Fig. S4A). Reduced energy intake, along with increased activity levels at increasing workloads, led to a flattening of the somatic growth curve with lower body mass at high workloads (*F*_1,2412_ = 67.00, *p* < 0.0001) and at low ambient temperatures (*p* < 0.05; Suppl. Figs. S1 and S4E, S4F). Furthermore, reducing the ambient temperature from 21° C to 10° C at ad libitum conditions caused only a slight reduction (0.4° C) in average (21° C: M = 38.06, SD = 0.03; 10° C: M = 37.67, SD = 0.20), minimum (21° C: M = 37.59, SD = 0.04; 10° C: M = 37.15, SD = 0.26), and maximum body temperature (21° C: M = 38.58, SD = 0.05; 10° C: M = 38.20, SD = 0.16). This finding shows that body temperature is rather stable at different ambient temperatures, while O_2_ consumption is increased at low ambient temperatures (Daan and Slopsema, 1978). These data confirm that the work-for-food protocol and ambient temperature manipulations can be used independently as tools to induce a negative energy balance in voles.

### A Negative Energy Balance Enhances Ultradian Activity

**Figure 1.**
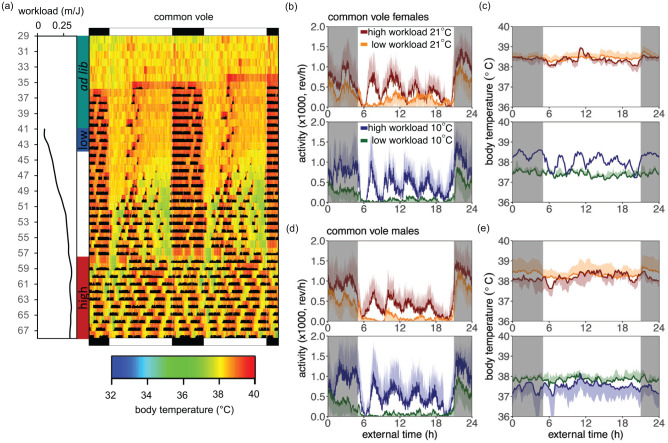
High workload causes a switch from nocturnal circadian activity and body temperature rhythms to ultradian patterns in common voles. (a) Representative double-plotted actogram and body temperature patterns of a male at gradually increasing workload at 21° C. Black lines indicate workload—the distance in meters that a vole had to run to obtain a 1-J food reward. Colors indicate body temperature ranging from blue (32° C) to red (40° C). Light-dark cycles are plotted on the top and bottom of actograms. Ad libitum, low and high workload conditions are indicated next to actograms. (b, c) Wheel running activity and core body temperature profiles for females and (d, e) males at high or low workload at 21° C or 10° C. Activity data are shown in 1-min bins. Core body temperature data are shown in 10-min bins. Five-day average activity profiles at the end of the work-for-food procedure are shown for each group. Data are presented as mean ± SD.

**Figure 2.**
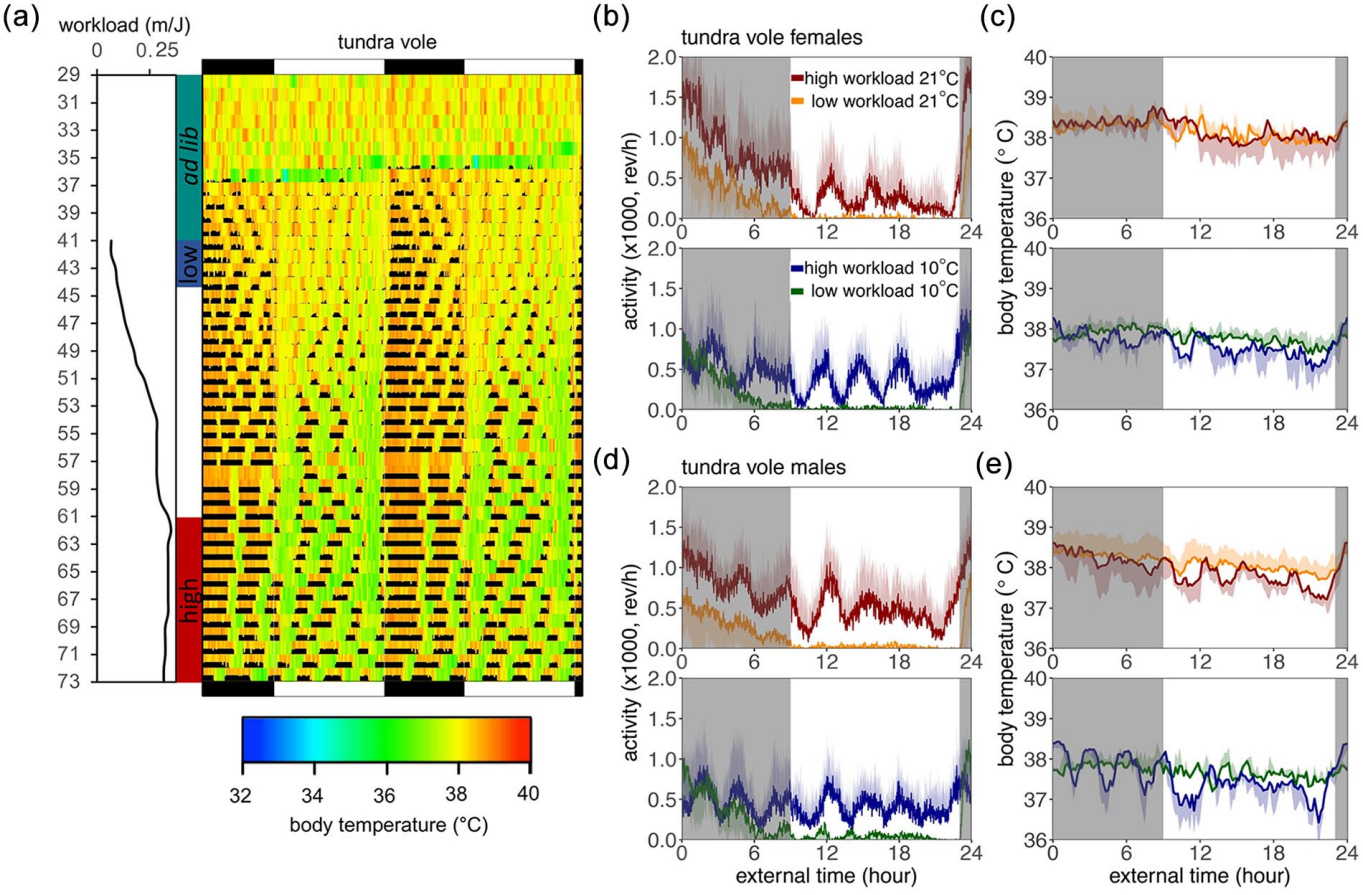
High workload causes a switch from nocturnal circadian activity and body temperature rhythms to ultradian patterns in tundra voles. a) Representative double-plotted actogram and body temperature patterns of a male at gradually increasing workload at 21° C. Black lines indicate workload—the distance in meters that a vole had to run to obtain a 1-J food reward. Colors indicate body temperature ranging from blue (32° C) to red (40° C). Light-dark cycles are plotted on the top and bottom of actograms. Ad libitum, low and high workload conditions are indicated next to actograms. (b, c) Wheel running activity and core body temperature profiles for females and (d, e) males at high or low workload at 21° C or 10° C. Activity data are shown in 1-min bins. Core body temperature data are shown in 10-min bins. Five-day average activity profiles at the end of the work-for-food procedure are shown for each group. Data are presented as mean ± SD.

**Figure 3.**
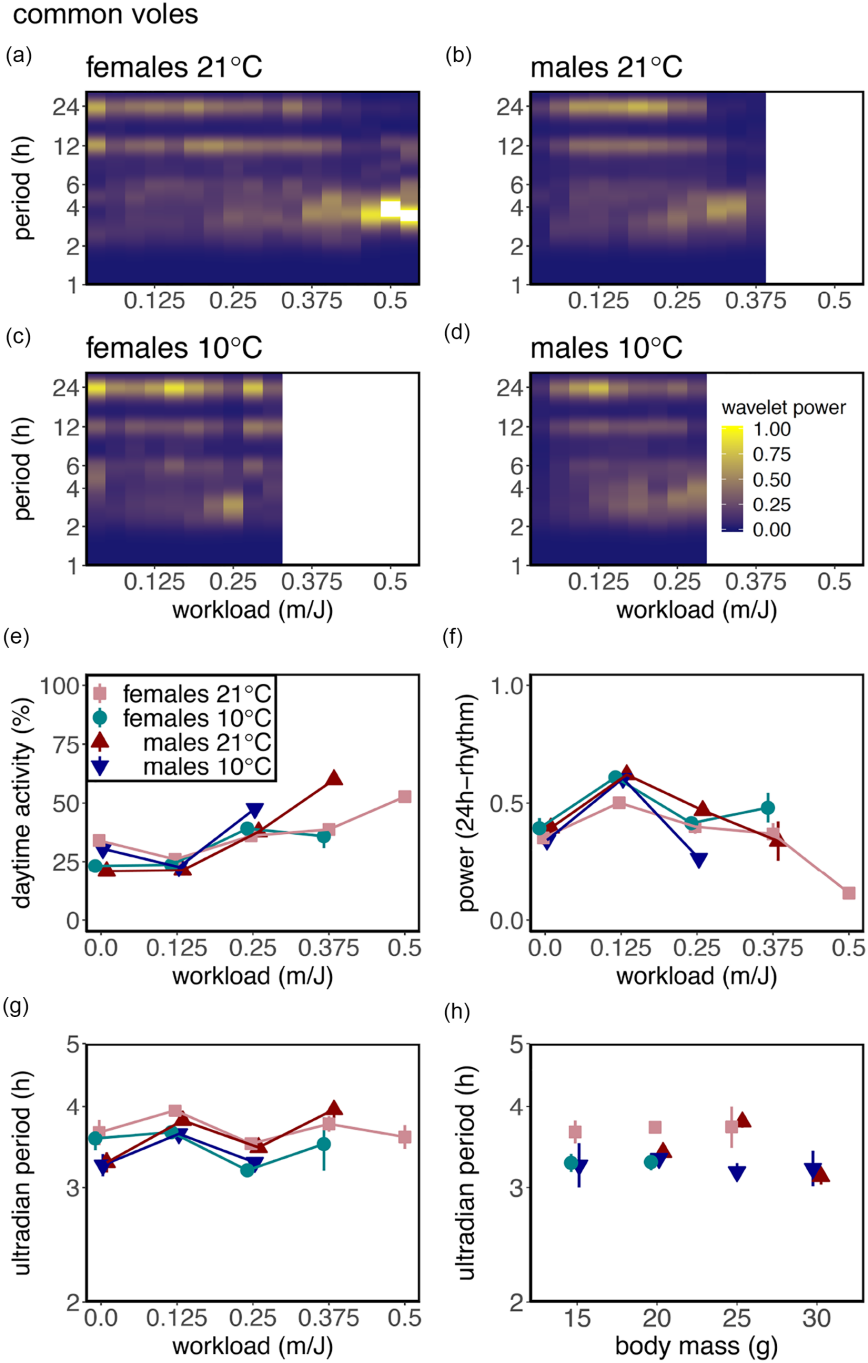
Energetic challenge causes enhancement of ultradian rhythmicity in common voles. Average wavelet power for the period of the activity rhythm related to workload in colored heatmaps for females at (a) 21° C or (c) 10° C and for males at (b) 21° C or (d) 10° C. Yellow indicates high wavelet power for a certain period and blue indicates low wavelet power. (e) % diurnal activity, (f) power of the 24-h rhythm, and (g) ultradian period related to workload and to (h) body mass. Workload indicates the distance in meters that a vole had to run to obtain a 1-J food reward. Data are presented as mean ± standard error of the mean. Significant effects of workload (wl), body mass (bm), temperature (temp), sex, and interactions are indicated by asterisks: *p < 0.05, **p < 0.01, ***p < 0.001. Daytime activity: wl***, wl × temp**, wl × sex***, wl × temp × sex***; power 24-h rhythm: ns; ultradian period: temp*; ultradian period: bm × temp***. Statistic results for repeated-measures linear mixed-effects models can be found in Supplementary Table S1.

**Figure 4.**
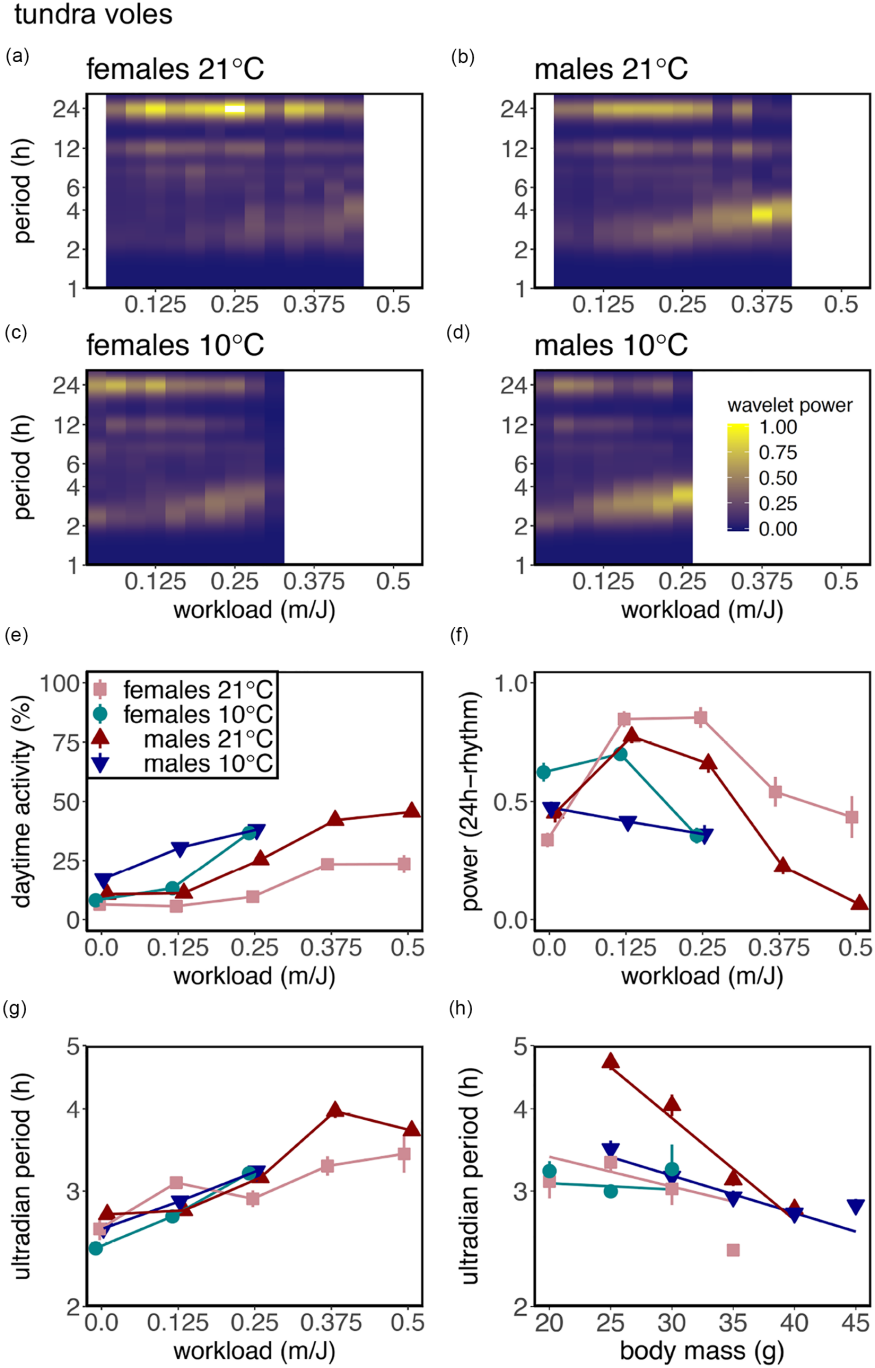
Energetic challenge causes lengthening of the ultradian period in tundra voles. Average wavelet power for the period of the activity rhythm related to workload in colored heatmaps for females at (a) 21° C or (c) 10° C and for males at (b) 21° C or (d) 10° C. Yellow indicates high wavelet power for a certain period and blue indicates low wavelet power. (e) % diurnal activity, (f) power of the 24-h rhythm, and (g) ultradian period related to workload and to (h) body mass. Workload indicates the distance in meters that a vole had to run to obtain a 1-J food reward. Data are presented as mean ± standard error of the mean. Significant effects of workload (wl), body mass (bm), temperature (temp), sex, and interactions are indicated by asterisks: *p < 0.05, **p < 0.01, ***p < 0.001.Daytime activity: wl***, temp***, sex***, wl × temp***, wl × sex***, wl × temp × sex*; power 24-h rhythm: temp**, sex*, wl × temp***, wl × temp x sex*; ultradian period: wl***, wl × temp × sex*; ultradian period: body mass***, sex**, bm × sex*. Statistic results for repeated-measures linear mixed-effects models can be found in Supplementary Table S1.

The finding that increasing workload caused an increase in total activity (Suppl. Fig. S4A and S4B) is related to a change in both phase and period of the activity rhythm. Voles at increasing workload show a robust increase in % daytime activity (common voles: *F*_1,2044_ = 226.52, *p* < 0.0001; tundra voles: *F*_1,2152_ = 714.56, *p* < 0.0001; Figs. 3e and 4e), but not at the expense of nocturnal activity (Suppl. Fig. S6). This is caused by the effect that increasing workload gradually changed nocturnal circadian activity and body temperature rhythms to ultradian rhythms spread out over the 24-h day (Figs. 1 and 2 and Suppl. Figs. S2 and S3). Interestingly, in tundra voles, % daytime activity was enhanced at 10° C (*F*_1,61_ = 21.77, *p* < 0.0001; Fig. 4e). Morlet wavelet time-series analysis revealed that the power of the 24-h rhythm increases from workload 0-0.125 m/J, whereas the power of the 24-h rhythm decreases from workload >0.125 m/J onward (Figs. 3f and 4f). Activity onset at lights-off and activity offset at lights-on are observed in the average activity profiles, even when high-amplitude ultradian rhythms are observed (Figs. 1 and 2 and Suppl. Figs. S2 and S3). However, at individual level, voles displayed unstable ultradian rhythm synchronization (Fig. 1a).

### Period of the Ultradian Rhythm Depends on Metabolic Demands

To investigate whether the ultradian period length is affected by the severity of the negative energy balance, spectral analyses have been performed. Heatmaps of wavelet analysis revealed that at low workloads, 24-h period activity patterns are most pronounced, whereas at high workloads 2- to 5-h periods appear (Figs. 3a-3d and 4a-4d). Filtered ultradian rhythms (i.e., period <5 h) show that a negative energy balance is lengthening the ultradian period with approximately 2 h in tundra voles at high workload (*F*_1,1857_ = 197.99, *p* < 0.0001; Fig. 4g). In contrast, ultradian period in common voles is not significantly affected by workload or body mass (workload: *F*_1,2058_ = 0.95, ns; Fig. 3g; body mass: *F*_1,2058_ = 3.42, ns; Fig. 3h). In tundra voles, the sex-dependent negative relationship between the period of the short-term activity rhythm and body mass indicates that metabolic status is involved in the regulation of the ultradian rhythm (tundra voles: *F*_1,1025_ = 10.98, *p* < 0.003; Fig. 4h). In tundra voles, an age effect is observed only in females at 21°C (Suppl. Fig. S5B; *F*_1,76_ = 9.26, *p* < 0.004).

## Discussion

Our results show that the expression of circadian and ultradian behavior is plastic and can be shaped by energetic demands. The power of ultradian activity rhythm increases when energetically challenged under long photoperiods, while the circadian component of running wheel activity decreases (Figs. 1-4). Furthermore, the period of the ultradian rhythmicity in our voles is related to energy balance indicators such as workload, ambient temperature, and body mass (Fig. 3f-3h and 4f-4h). Ultradian periods were longer at high workloads in tundra voles, but not in common voles. This indicates that in common voles, ultradian rhythmicity is driven by an oscillator, whereas in tundra voles, ultradian rhythmicity is primarily dependent on metabolic status. A negative energy balance induced by food scarcity has different effects on ultradian rhythmicity than a negative energy balance induced by temperature manipulations. These observations confirm that both ultradian and circadian rhythm expression in voles are inversely related to metabolic status. Our findings indicate that a negative energy balance overrules other environmental components including long photoperiods to enhance ultradian rhythms. Here, voles were photoperiodical spring programmed (i.e., gestated and raised to weaning under SP), at which breeding would occur. Maternal photoperiodic programming may induce opposite reproductive and metabolic status in autumn-programmed animals (Sáenz de Miera et al., 2017), in which the greatest energy demands are expected, and therefore ultradian rhythms may be prominent.

House mice can reduce energy expenditure by being active during daytime (i.e., the warmest part of the day), while resting and showing torpor during the night (i.e., coldest part of the day) (Hut et al., 2012; van der Vinne et al., 2015b). The increase in % diurnal activity when having a negative energy balance (Figs. 3e and 4e and Suppl. Fig. S6) is in line with earlier studies in mice (Hut et al., 2011; van der Vinne et al., 2014). In contrast, voles display circadian arrhythmicity at high workloads, resulting in activity that is almost equally distributed between day and night (Figs. 3e and 4e), whereas house mice are primarily active (>50% diurnal) during daytime at high workloads. This finding along with the absence of torpor in our energetically challenged voles (Figs. 1 and 2) suggests that in voles, the behavioral response to energetically demanding conditions will generate smaller energetic benefits compared with house mice, suggesting that ultradian vole strategy may have other benefits specific for *Microtus* species.

In voles, nocturnal activity may be beneficial to prevent predation by avian diurnal birds of prey like the kestrel (Daan et al., 1982; Masman et al., 1988; Rijnsdorp et al., 1981). However, when food resources become limited and temperatures drop, a negative energy balance arises, which increases metabolic demands. Under these circumstances, their foraging strategy will change by displaying ultradian activity (Figs. 1-4). This leads to an increased period of time to gather enough food to survive. By means of triggering ultradian activity instead of shifting from nocturnal to diurnal, like house mice do (Hut et al., 2011), individual predation risk of voles will be minimized (Daan and Slopsema, 1978; Gerkema and Verhulst, 1990). Annual changes in food availability will change the metabolic status of voles, which may cause seasonal fluctuations in the amplitude of ultradian rhythmicity as also has been observed in voles at populational level (Daan and Slopsema, 1978; Halle, 1995).

Phase-setting of ultradian activity appeared both at lights-on (activity offset) and at lights-off (activity onset) (Figs. 1 and 2), which previously has been demonstrated in voles (Halle, 1995; Lehmann and Sommersberg, 1980). This observation is in agreement with the “two-oscillator hypothesis” described by Pittendrigh (1960) and Daan and Berde (1978), and illustrates coupling of the ultradian and circadian systems, which allows seasonal adjustment of ultradian rhythms. However, in some ultradian animals, the circadian component of running wheel activity and synchronization to the light-dark cycle is completely eliminated (Fig. 1a). This finding suggests that the ultradian component overrules the circadian component under high energetic demands, which allows activity patterns to adapt to specific ecological conditions. In contrast, ultradian activity and physiological patterns have been observed in the absence of energetic challenges (Lewis and Curtis, 2016; van der Veen et al., 2006), which may be the result of voles having no access to a running wheel (van der Veen et al., 2006). Furthermore, the power of the 24-h rhythm increases early in the protocol (Figs. 3f and 4f), which may be caused by habituation to having access to a running wheel (van der Veen et al., 2006). Further increase in workload caused a strong reduction in the power of the 24-h rhythm (Figs. 3f and 4f).

A negative energy balance lengthens the ultradian period in tundra voles (Fig. 4g), but not in common voles (Fig. 3g). Heatmaps of male common voles (Fig. 3b and 3d) indicate a potential ultradian period change related to workload. However, this effect is not reflected in our analysis (Fig. 3g). This may be the result of the ultradian period around workload 0.125 m/J, which perhaps shows an interaction with the 24-h rhythm. In tundra voles, the period of the short-term activity rhythm depends on energy balance (Fig. 4) and supports the hypothesis that short-period rhythms are causally related to metabolic needs.

The lack of the positive relationship between ultradian period and the severity of the negative energy balance in common voles is in agreement to what has been observed in an earlier study (Gerkema and van der Leest, 1991). In this study, food deprivation did not change ultradian period (Gerkema and van der Leest, 1991). The finding that ultradian patterns arise earlier at low temperatures (Figs. 3 and 4) confirms that a negative energy balance enhances ultradian rhythmicity. A positive relationship between ambient temperature and period has previously been found (Daan and Slopsema, 1978). The observation that period length is decreased at low temperature (Figs. 3 and 4) shows that a negative energy balance induced by food scarcity has different effects on ultradian period than a negative energy balance induced by temperature manipulations. Part of the explanation might be that induction of a negative energy balance by the work-for-food procedure requires increased activity to secure food. For this reason, there is limited time to rest between activity bouts, leading to increased ultradian periods at high workloads. Induction of a negative energy balance via temperature allows animals to eat fast and after a short period move back to their insulating burrow to rest and save energy.

Females are more resistant to high workloads without losing too much energy (Figs. 3 and 4). In general, females are smaller than males, and therefore need less food to retain the same energy balance. Furthermore, communal living in female voles may alter energy demands when food is scarce at low temperatures (Dobly, 2009; Hayes and Solomon, 2004). However, our experimental design using running wheels required individual housing without nesting material, which may result in different energy demands from what is expected under natural circumstances. Therefore, our results should be interpreted with caution. The observed longer period in common voles at low workload compared with tundra voles at low workload (Figs. 3g and 4g) may be partly attributed to the lower body mass of common voles (Suppl. Fig. S1). An additional explanation might be the different photoperiods common and tundra voles were exposed to for other reasons not related to this article.

Altogether, our findings reveal different systems for ultradian regulation in common and tundra voles. In common voles, ultradian rhythmicity may be driven by an oscillator, which hints at synchronization of ultradian activity among individuals to reduce individual predation risk by safety in numbers (Daan and Slopsema, 1978). Tundra voles are common at high latitudes, where they live in tunnels covered by snow in winter and early spring, which protects them from predators. For this reason, an oscillator for ultradian rhythmicity may be of less importance in tundra voles. In tundra voles, ultradian rhythmicity depends on food intake, which indicates that the ultradian system of this species is aimed at optimal food digestion. This is in line with the environment where food scarcity potentially plays an important role for behavioral adaptation. In conclusion, this study shows that the balance between circadian and ultradian expression of behavioral rhythmicity is determined by energy balance, confirming that voles have a highly flexible timing mechanism for activity, which allows behavioral adaptation to specific environmental conditions.

## Supplemental Material

sj-pdf-1-jbr-10.1177_07487304211005640 – Supplemental material for Negative Energy Balance Enhances Ultradian Rhythmicity in Spring-Programmed Voles Click here for additional data file. Supplemental material, sj-pdf-1-jbr-10.1177_07487304211005640 for Negative Energy Balance Enhances Ultradian Rhythmicity in Spring-Programmed Voles by Laura van Rosmalen and Roelof A. Hut in Journal of Biological Rhythms
