# Supplementary material for: Negative Energy Balance Enhances Ultradian Rhythmicity in Spring-Programmed Voles
Source: J Biol Rhythms. 2021 Apr 20;36(4):359–68. doi: 10.1177/07487304211005640 (PMC8276337; doi:10.1177/07487304211005640)
Supplement: sj-pdf-1-jbr-10.1177_07487304211005640 – Supplemental material for Negative Energy Balance Enhances Ultradian Rhythmicity in Spring-Programmed Voles [file sj-pdf-1-jbr-10.1177_07487304211005640.pdf]

Supplementary information

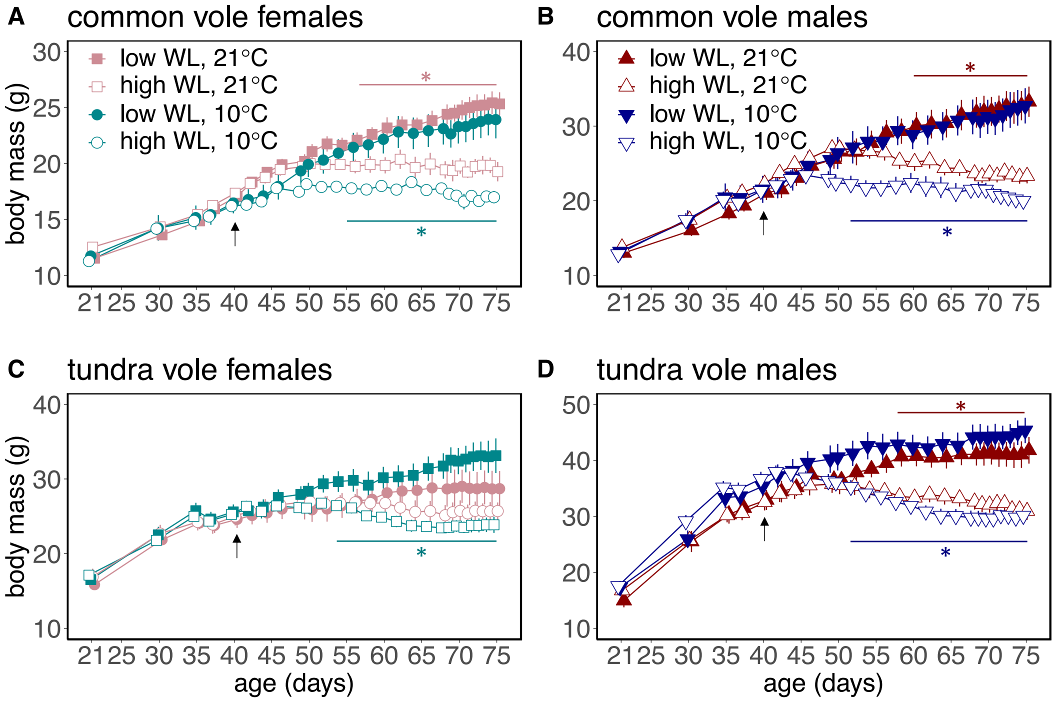

Figure S1. Body mass growth curves of voles under different workload and temperature conditions. Growth curves for (A) common vole females, (B) common vole males, (C) tundra vole females and (D) tundra vole males. The arrow indicates the start of the 'work-for-food' protocol. Data are presented as means ± SEM. Significant differences between workloads are indicated by an asterisk ($p < 0.05$). Statistic results for linear mixed-effects models can be found in table S1.

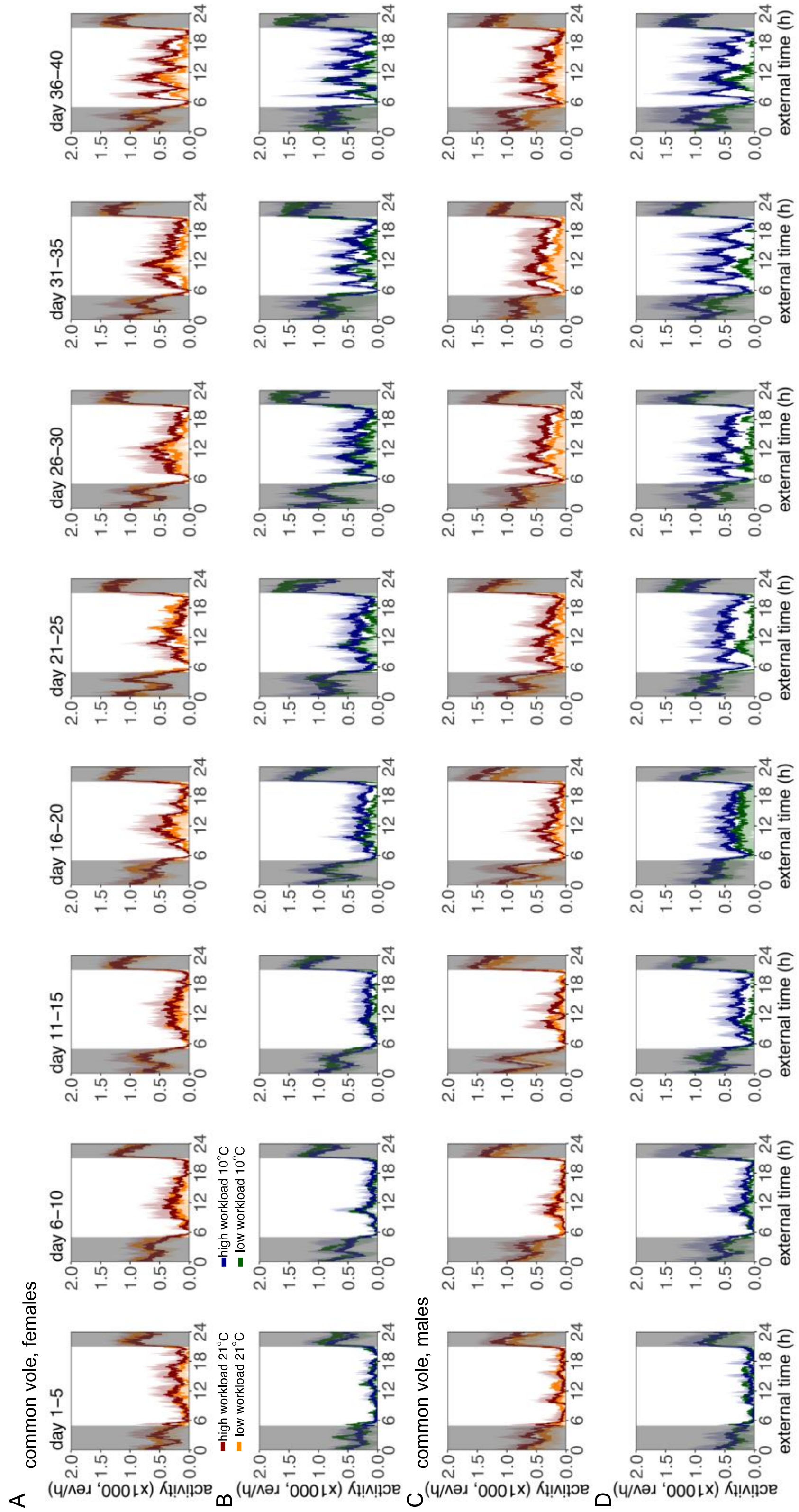

Figure S2. Running wheel activity profiles for common voles under high- and low workload regimes. 5-day average locomotor activity profiles for (A) females at 21°C, (B) females at 10°C, (C) males at 21°C, (D) males at 10°C at high- or low workload regimes. Activity data is shown in 1-minute bins. Data are presented as means ± SD.

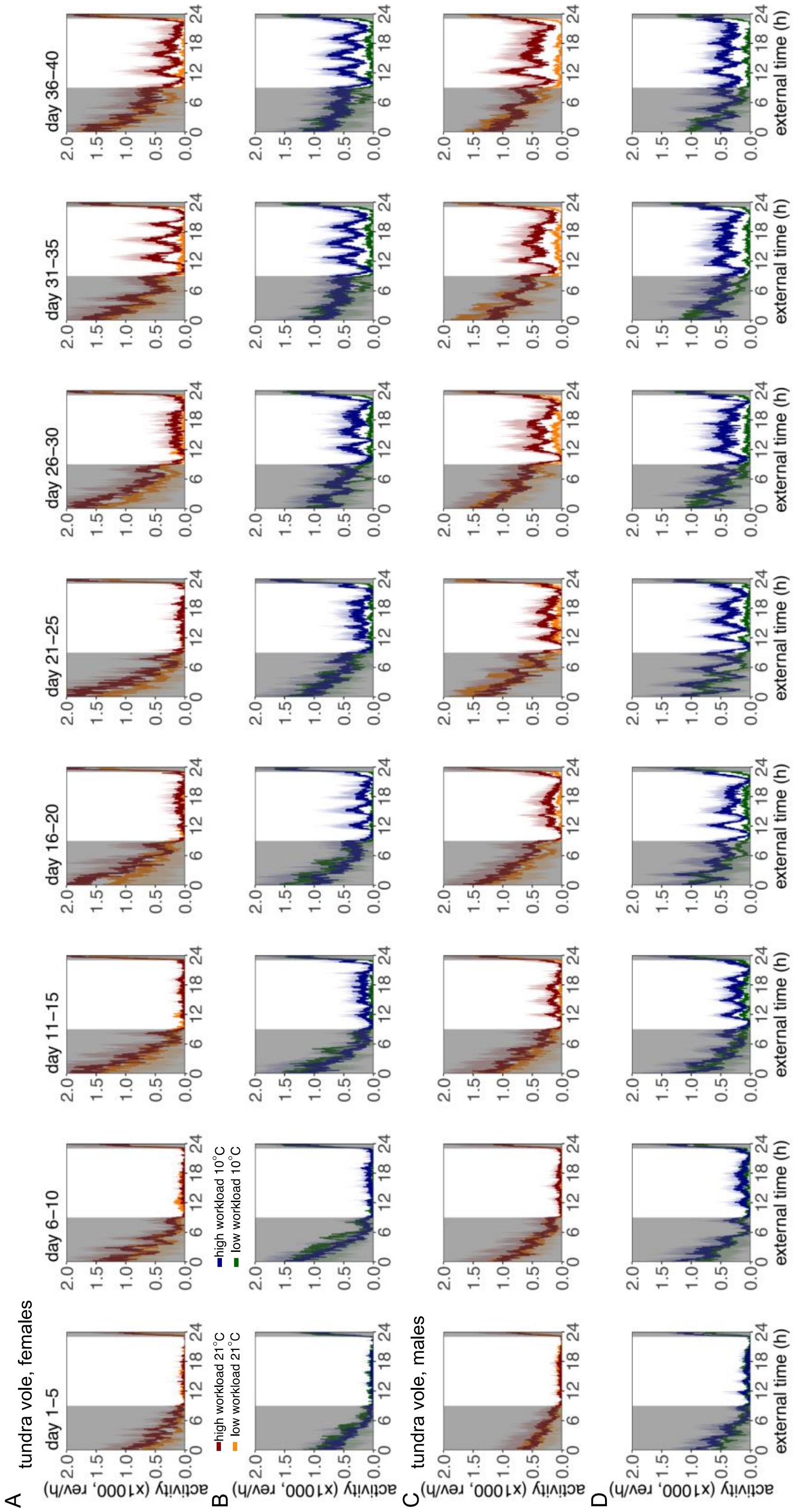

Figure S3. Running wheel activity profiles for tundra voles under high- and low workload regimes. 5-day average locomotor activity profiles for (A) females at 21°C, (B) females at 10°C, (C) males at 21°C, (D) males at 10°C at high- or low workload regimes. Activity data is shown in 1-minute bins. Data are presented as means ± SD.

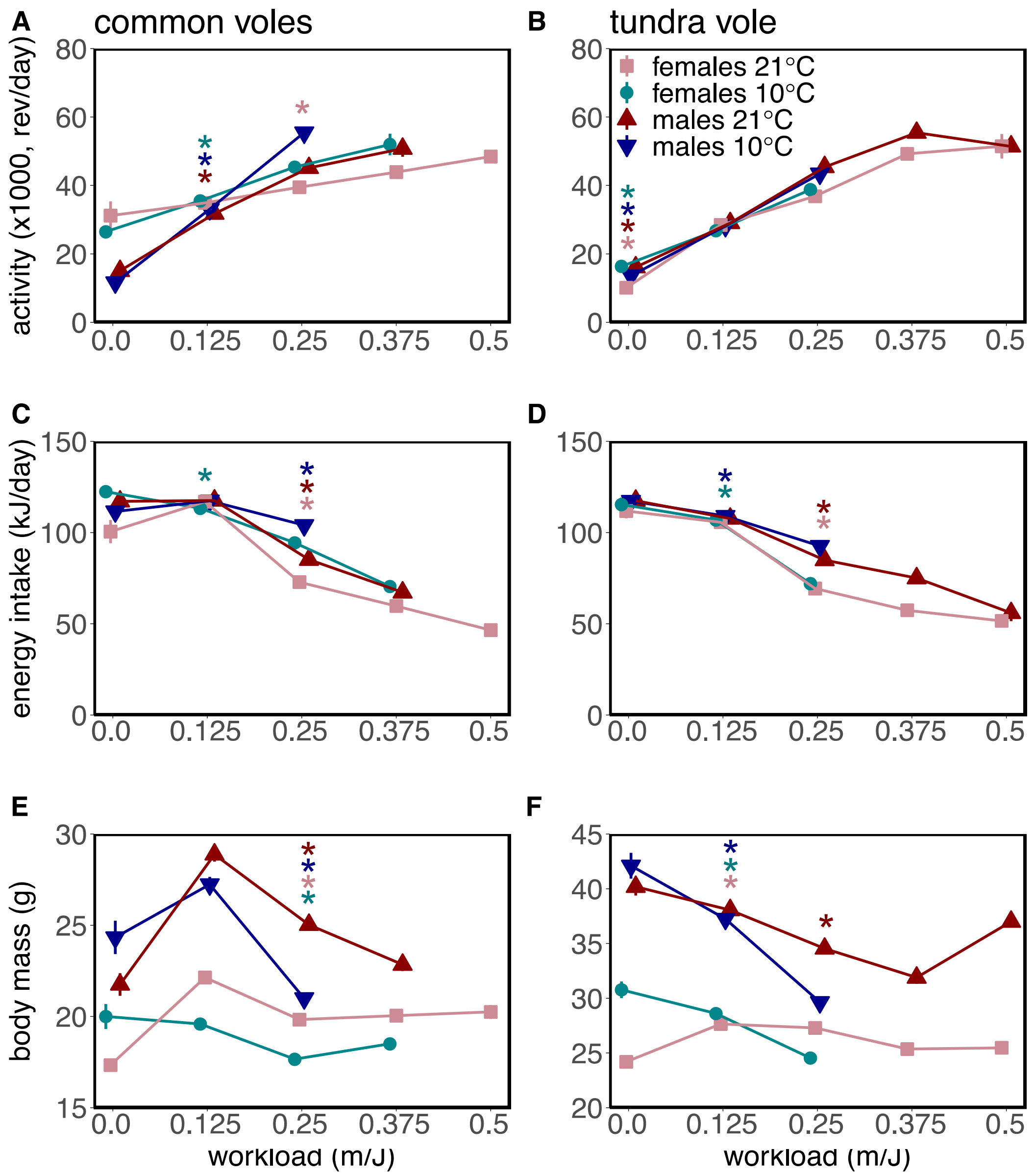

Figure S4. Total daily activity, energy intake and body mass in relation to workload. (A, B) Daytime activity levels, (C, D) energy intake and (E, F) body mass related to workload is depicted for common and tundra voles respectively at 21°C or 10°C. Workload indicates the distance in m that a vole had to run to obtain a 1 Joule food reward. Data are presented as means ± SEM. Asterisks indicate workload at which activity, energy intake or body mass become statistically different from baseline for each group. Statistic results for linear mixed-effects models can be found in table S1.

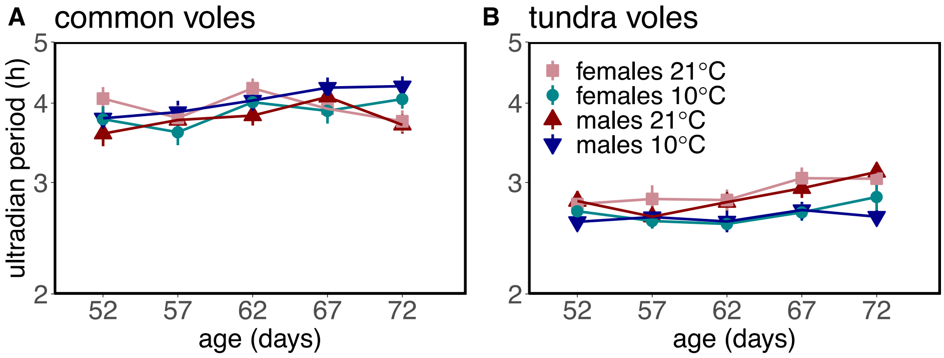

Figure S5. Period of the ultradian rhythm in relation to time, workload and body mass. Period in relation to age (constant low workload) for (A) common and (B) tundra voles. Data are presented as means ± SEM. Statistic results for linear mixed-effects models can be found in table S1.

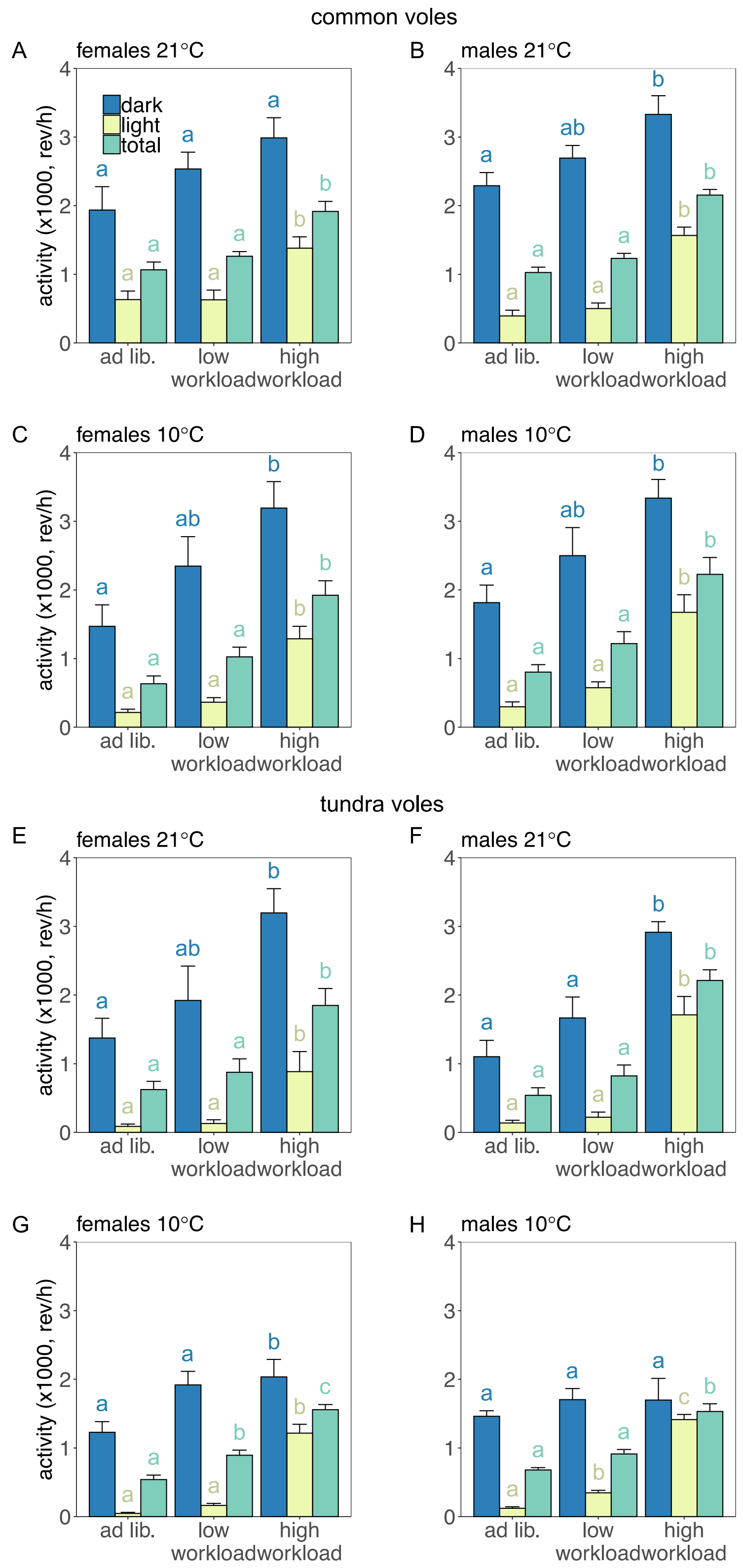

Figure S6. Total, dark and light running wheel activity levels at different workloads. Graphs depict night (blue), day (yellow) and total activity (green) levels per hour at *ad libitum*, low workload or high workload conditions for (A) common vole females at 21°C, (B) common vole males at 21°C, (C) common vole females at 10°C, (D) common vole males at 10°C, (E) tundra vole females at 21°C, (F) tundra vole males at 21°C, (G) tundra vole females at 10°C, (H) tundra vole males at 10°C. Data is presented as means ± SEM. Different letters above bars indicate significant different groups for either dark, light or total activity ($p < 0.05$).

Table S1. Statistics for linear mixed-effects models. Variables with non-significant *p*-values were omitted for final models.

**Fig. 3E, 4E**	**daytime activity**					
	Df	*F*	*p*			
intercept	1, 4197	622.0330	< 0.0001***			
workload (wl)	1, 4197	799.2529	< 0.0001***			
temperature (temp)	1, 123	13.2798	< 0.0005***			
sex	1, 123	9.5699	< 0.003**			
species	1, 123	31.0125	< 0.0001***			
wl x temp	1, 4197	48.5335	< 0.0001***			
wl x sex	1, 4197	54.3523	< 0.0001***			
temp x sex	1, 123	1.9915	< 0.16			
wl x species	1, 4197	26.0548	< 0.0001***			
temp x species	1, 123	9.7696	< 0.003**			
sex x species	1, 123	7.2908	< 0.008**			
wl x temp x sex	1, 4197	23.7711	< 0.0001***			
wl x temp x species	1, 4197	20.0098	< 0.0001***			
wl x sex x species	1, 4197	9.0737	< 0.003**			
**Fig. 3E, 4E**	**daytime activity (common voles)**			**daytime activity (tundra voles)**		
	Df	*F*	*p*	Df	*F*	*p*
intercept	1, 2044	440.9053	< 0.0001***	1, 2152	193.6851	< 0.0001***
workload (wl)	1, 2044	226.5217	< 0.0001***	1, 2152	714.5610	< 0.0001***
temperature (temp)	1, 61	0.3828	< 0.54	1, 61	21.7704	< 0.0001***
sex	1, 61	0.5140	< 0.48	1, 61	15.3617	< 0.0003***
wl x temp	1, 2044	7.5470	< 0.007**	1, 2152	84.1589	< 0.0001***
wl x sex	1, 2044	40.8688	< 0.0001***	1, 2152	16.1292	< 0.0002***
temp x sex	1, 61	1.1354	< 0.29	1, 61	0.6554	< 0.43
wl x temp x sex	1, 2044	20.2279	< 0.0001***	1, 2152	5.0659	< 0.03*
**Fig. 3F, 4F**	**power 24h-rhythm**					
	Df	*F*	*p*			
intercept	1, 4496	691.1378	< 0.0001***			
workload (wl)	1, 4496	0.1245	< 0.73			
temperature (temp)	1, 122	4.3623	< 0.04*			
sex	1, 122	2.8801	< 0.09			
species	1, 122	6.6988	< 0.02*			
wl x temp	1, 4496	6.9363	< 0.009**			
wl x sex	1, 4496	0.5833	< 0.45			
temp x sex	1, 122	2.2755	< 0.14			
wl x species	1, 4496	3.5377	< 0.07			
temp x species	1, 122	4.9624	< 0.03*			
sex x species	1, 122	2.4380	< 0.13			
wl x temp x sex	1, 4496	2.2502	< 0.14			
wl x temp x species	1, 4496	15.0336	< 0.0002***			
wl x sex x species	1, 4496	0.0215	< 0.89			
temp x sex x species	1, 122	0.0304	< 0.87			
wl x temp x sex x species	1, 4496	4.0618	< 0.05*			
**Fig. 3F, 4F**	**power 24h-rhythm (common vole)**			**power 24h-rhythm (tundra vole)**		
	Df	*F*	*p*	Df	*F*	*p*
intercept	1, 2263	300.28562	< 0.0001***	1, 2233	390.9739	< 0.0001***
workload (wl)	1, 2263	1.14376	< 0.29	1, 2233	2.0625	< 0.16
temperature (temp)	1, 63	0.00628	< 0.94	1, 59	8.5920	< 0.005**
sex	1, 63	0.18619	< 0.67	1, 59	5.4822	< 0.03*
wl x temp	1, 2263	0.33827	< 0.57	1, 2233	16.0991	< 0.0002***
wl x sex	1, 2263	1.72905	< 0.19	1, 2233	0.5187	< 0.48
temp x sex	1, 63	0.79705	< 0.38	1, 59	0.5889	< 0.45
wl x temp x sex	1, 2263	0.02162	< 0.89	1, 2233	5.1054	< 0.03*
**Fig. 3G, 4G**	**ultradian period**					
	Df	*F*	*p*			
intercept	1, 3915	49357.18	< 0.0001***			
workload (wl)	1, 3915	66.55	< 0.0001***			

temperature (temp)	1, 122	6.60	< 0.02*			
sex	1, 122	0.72	< 0.40			
species	1, 122	95.33	< 0.0001***			
wl x temp	1, 3915	0.97	< 0.33			
wl x sex	1, 3915	8.84	< 0.004**			
temp x sex	1, 3915	1.38	< 0.25			
wl x species	1, 3915	91.00	< 0.0001***			
temp x species	1, 122	1.69	< 0.20			
sex x species	1, 122	1.82	< 0.18			
wl x temp x sex	1, 3915	0.10	< 0.76			
wl x temp x species	1, 3915	0.97	< 0.33			
wl x sex x species	1, 3915	0.00	< 0.96			
temp x sex x species	1, 122	0.03	< 0.87			
wl x temp x sex x species	1, 3915	8.54	< 0.004**			
**Fig. 3G, 4G**	**ultradian period (common voles)**			**ultradian period (tundra voles)**		
	Df	*F*	*p*	Df	*F*	*p*
intercept	1, 2058	18464.560	< 0.0001***	1, 1857	35580.69	< 0.0001***
workload (wl)	1, 2058	0.004	< 0.95	1, 1857	197.99	< 0.0001***
temperature (temp)	1, 63	4.187	< 0.05*	1, 59	0.11	< 0.74
sex	1, 63	1.353	< 0.25	1, 59	0.64	< 0.43
wl x temp	1, 2058	1.837	< 0.18	1, 1857	0.02	< 0.90
wl x sex	1, 2058	1.408	< 0.24	1, 1857	1.02	< 0.32
temp x sex	1, 63	0.428	< 0.52	1, 59	1.14	< 0.30
wl x temp x sex	1, 2058	3.366	< 0.07	1, 1857	6.02	< 0.02*
**Fig. 3H, 4H**	**ultradian period**					
	Df	*F*	*p*			
intercept	1, 2181	43024.31	< 0.0001***			
body mass (bm)	1, 2181	32.98	< 0.0001***			
temperature (temp)	1, 119	8.80	< 0.004**			
sex	1, 119	2.45	< 0.12			
species	1, 119	48.83	< 0.0001***			
bm x temp	1, 2181	2.45	< 0.12			
bm x sex	1, 2181	4.28	< 0.04*			
bm x species	1, 2181	17.66	< 0.0001***			
temp x species	1, 119	1.04	< 0.32			
sex x species	1, 119	11.31	< 0.002**			
bm x temp x sex	1, 2181	11.01	< 0.001***			
bm x sex x species	1, 2181	5.31	< 0.03*			
**Fig. 3H, 4H**	**ultradian period (common voles)**			**ultradian period (tundra voles)**		
	Df	*F*	*p*	Df	*F*	*p*
intercept	1, 1154	19823.093	< 0.0001***	1, 1025	22840.886	< 0.0001***
body mass (bm)	1, 1154	3.425	< 0.06	1, 1025	21.599	< 0.0001***
temperature (temp)	1, 59	2.421	< 0.13	1, 58	3.425	< 0.07
sex	1, 59	3.234	< 0.08	1, 58	8.942	< 0.004**
bm x temp	1, 1154	12.716	< 0.0005***	1, 1025	0.702	< 0.41
bm x sex	1, 1154	0.008	< 0.94	1, 1025	10.977	< 0.002**
temp x sex	1, 59	0.003	< 0.96	1, 58	0.479	< 0.50
bm x temp x sex	1, 1154	0.774	< 0.38	1, 1025	0.333	< 0.56
**Fig. S4A, B**	**total activity**					
	Df	*F*	*p*			
intercept	1, 4199	3012.580	< 0.0001***			
workload (wl)	1, 4199	3859.233	< 0.0001***			
temperature (temp)	1, 123	0.289	< 0.60			
sex	1, 123	2.459	< 0.12			
species	1, 123	23.742	< 0.0001***			
wl x temp	1, 4199	55.181	< 0.0001***			
wl x sex	1, 4199	113.849	< 0.0001***			
wl x species	1, 4199	14.320	< 0.0003***			
temp x species	1, 123	1.209	< 0.28			
sex x species	1, 123	0.753	< 0.39			

wl x temp x species	1, 4199	77.539	< 0.0001***			
wl x sex x species	1, 4199	4.696	< 0.04*			
**Fig. S4A, B**	**total activity (common vole)**			**total activity (tundra vole)**		
	Df	*F*	*p*	Df	*F*	*p*
intercept	1, 2044	1982.9563	< 0.0001***	1, 2152	1096.8576	< 0.0001***
workload (wl)	1, 2044	1866.3732	< 0.0001***	1, 2152	1991.3810	< 0.0001***
temperature (temp)	1, 61	2.2614	< 0.14	1, 61	0.3429	< 0.57
sex	1, 61	0.6407	< 0.43	1, 61	1.4299	< 0.24
wl x temp	1, 2044	161.7185	< 0.0001***	1, 2152	4.2039	< 0.05*
wl x sex	1, 2044	72.6706	< 0.0001***	1, 2152	23.9135	< 0.0001***
temp x sex	1, 61	0.1799	< 0.68	1, 61	0.0536	< 0.82
wl x temp x sex	1, 2044	1.1381	< 0.29	1, 2152	2.5472	< 0.12
**Fig. S4C, D**	**energy intake**					
	Df	*F*	*p*			
intercept	1, 4413	7066.901	< 0.0001***			
workload (wl)	1, 4413	1308.918	< 0.0001***			
temperature (temp)	1, 137	1.651	< 0.21			
sex	1, 137	6.305	< 0.02*			
species	1, 137	10.875	< 0.002**			
wl x temp	1, 4413	10.226	< 0.002**			
wl x sex	1, 4413	18.074	< 0.0001***			
temp x sex	1, 4413	0.444	< 0.51			
wl x species	1, 4413	0.329	< 0.57			
temp x species	1, 137	0.578	< 0.45			
sex x species	1, 137	0.391	< 0.54			
wl x temp x sex	1, 4413	4.375	< 0.04*			
wl x temp x species	1, 4413	62.451	< 0.0001***			
wl x sex x species	1, 4413	9.831	< 0.002**			
**Fig. S4C, D**	**energy intake (common vole)**			**energy intake (tundra vole)**		
	Df	*F*	*p*	Df	*F*	*p*
intercept	1, 2184	6376.627	< 0.0001***	1, 2228	2271.4033	< 0.0001***
workload (wl)	1, 2184	858.810	< 0.0001***	1, 2228	530.6548	< 0.0001***
temperature (temp)	1, 71	4.582	< 0.04*	1, 65	0.0292	< 0.87
sex	1, 71	3.879	< 0.05*	1, 65	3.1799	< 0.08
wl x temp	1, 2184	77.086	< 0.0001***	1, 2228	14.1535	< 0.0003***
wl x sex	1, 2184	0.172	< 0.68	1, 2228	21.1934	< 0.0001***
temp x sex	1, 71	0.175	< 0.68	1, 65	0.2373	< 0.63
wl x temp x sex	1, 2184	3.881	< 0.05*	1, 2228	0.1581	< 0.70
**Fig. S4E, F**	**body mass**					
	Df	*F*	*p*			
intercept	1, 2665	4852.990	< 0.0001***			
workload (wl)	1, 2665	25.958	< 0.0001***			
temperature (temp)	1, 124	1.248	< 0.27			
sex	1, 124	75.016	< 0.0001***			
species	1, 124	140.768	< 0.0001***			
wl x temp	1, 2665	3.213	< 0.08			
wl x sex	1, 2665	9.173	< 0.003**			
wl x species	1, 2665	59.712	< 0.0001***			
temp x species	1, 124	2.114	< 0.15			
sex x species	1, 124	11.064	< 0.002**			
wl x temp x species	1, 2665	6.739	< 0.01**			
wl x sex x species	1, 2665	13.279	< 0.0004***			
**Fig. S4E, F**	**body mass (common vole)**			**body mass (tundra vole)**		
	Df	*F*	*p*	Df	*F*	*p*
intercept	1, 1295	2038.8707	< 0.0001***	1, 1367	2744.7064	< 0.0001***
workload (wl)	1, 1295	89.5299	< 0.0001***	1, 1367	2.5119	< 0.12
temperature (temp)	1, 61	2.5161	< 0.12	1, 62	0.0227	< 0.89
sex	1, 61	24.1806	< 0.0001***	1, 62	57.1296	< 0.0001***
wl x temp	1, 1295	0.0685	< 0.80	1, 1367	7.8437	< 0.006**
wl x sex	1, 1295	0.6235	< 0.43	1, 1367	17.9278	< 0.0001***

temp x sex	1, 61	1.2330	< 0.27	1, 62	0.0037	< 0.96
wl x temp x sex	1, 1295	0.1515	< 0.70	1, 1367	4.0834	< 0.05*
**Fig. S5A, B**	**ultradian period**					
	Df	*F*	*p*			
intercept	1, 1276	26633.256	< 0.0001***			
age	1, 1276	13.058	< 0.0004***			
temperature (temp)	1, 56	1.400	< 0.25			
species	1, 56	147.814	< 0.0001***			
age x temp	1, 1276	0.079	< 0.78			
age x species	1, 1276	0.783	< 0.38			
temp x species	1, 56	2.589	< 0.12			
age x temp x species	1, 1276	8.946	< 0.003**			
**Fig. S5A, B**	**ultradian period (common vole)**			**ultradian period (tundra vole)**		
	Df	*F*	*p*	Df	*F*	*p*
intercept	1, 630	6967.591	< 0.0001***	1, 642	13484.257	< 0.0001***
age	1, 630	0.325	< 0.57	1, 642	11.990	< 0.0006**
temperature (temp)	1, 24	0.143	< 0.71	1, 28	3.172	< 0.09
sex	1, 24	2.098	< 0.17	1, 28	0.054	< 0.82
age x temp	1, 630	0.015	< 0.91	1, 642	4.548	< 0.04
age x sex	1, 630	5.201	< 0.03	1, 642	0.006	< 0.94
temp x sex	1, 24	1.001	< 0.34	1, 28	0.034	< 0.86
age x temp x sex	1, 630	1.368	< 0.25	1, 642	0.011	< 0.92
